# • Pancho trial (p53-adapted neoadjuvant chemotherapy for resectable esophageal cancer) completed—mutation rate of the marker higher than expected

**DOI:** 10.1007/s10353-018-0527-z

**Published:** 2018-06-11

**Authors:** Sonja Kappel-Latif, Johannes Zacherl, Michael Hejna, Maria Westerhoff, Dietmar Tamandl, Ahmed Ba-Ssalamah, Martina Mittlböck, Brigitte Wolf, Friedrich Wrba, Irene Kührer, Ursula Pluschnig, Sebastian F. Schoppmann, Reinhold Függer, Ronald Zwrtek, Karl Glaser, Josef Karner, Friedrich Längle, Etienne Wenzl, Rudolf Roka, Dietmar Öfner, Jörg Tschmelitsch, Michael Hold, Felix Keil, Michael Gnant, Daniela Kandioler, the Pancho trialists and for the Medical University of Vienna p53research group, D. Kandioler, J. Zacherl, M. Hejna, S. Schoppmann, M. Gnant, U. Pluschnig, I. Kührer, W. Klepetko, G. Prager, M. Riegler, C. Aigner, B. Teleky, S. Kappel-Latif, C. Bichler, B. Wolf, G. Werba, C. Sandurkov, D. Tamandl, A. Ba-Ssalamah, M. Uffmann, F. Wrba, M. Mittlböck, B. Niederle, R. Jakesz, F. Mühlbacher, J. Friedl, R. Kain, L. Brammen, K. Glaser, F. Berger, S. Brugger, S. Sporn, K. Strasser-Weipl, R. Fortelny, M. Essenther, A. Chott, J. Karner, S. Thalhammer, M. Klimpfinger, R. Roka, M. Schermann, M. Kees-Belyus, V. Sagaster, T. Grünberger, E. Bonner, M. Hold, M. Bernhart, S. Roka, C. Österreicher, V. Riegler, A. Nader, J. Haller, R. Zwrtek, P. Götzinger, M. Pober, T. Schenk, W. Guggenberger, R. Sedivy, M. Kitzwögerer, G. Heinz, M. Thür, F. Längle, I. Viragos-Toth, E. Frcena, H. Pourebrahim, E. Kristandl, W. Stiglbauer, R. Függer, F. Tomaselli, S. Metz, F. Moinfar, F. Keil, U. Kastner, H. Rabl, C. Tinchon, V. Odelga, N. Rapp, N. Eberhard, H. Kainz, M. Maderdonner, G. Leitner, J. Tschmelitsch, T. Eberl, H. J. Neumann, H. Weiß, J. Mühlmann, K. Weeber, G. Danko, D. Öfner, G. Mühlmann, M. Zitt, H. Maier, B. Heinke, W. Eisterer, N. Bergmann, E. Dablander, G. Mikuz, A. Brunner, E. Wenzl, A. Haid, K. Ammann, M. Knauer, A. Lang, B. Hartmann, M. Lercher-Lueger, F. Offner, B. Aberer, M. Hudec, M. Hohlagschwandtner, W. Trabe, P. Merz, V. Kadlecek, U. Smetana, D. Veit, T. Veit, D. Kerjaschki, O. Braun, R. Kuzmits, D. Kosak, W. Adolf, T. Kessler, C. Wüstinger, N. Neuhold, F. Beer, C. Freibauer, S. Naude, O. M. Braun, M. Mostegel, F. Pantucek, J. Feichtinger, W. Sega, H. Gogl, W. Höbling, R. Silye, E. Beck, H. Denk, G. Höfler, K. Lichtenegger, H. Rogatsch, S. Galowitsch, O. Dietze

**Affiliations:** 1 Division of General Surgery, Department of Surgery, Research Laboratories, Medical University of Vienna, Vienna, Austria; 2 Division of General Surgery, Department of Surgery and Comprehensive Cancer Center, Medical University of Vienna, Vienna, Austria; 3 Division of Oncology, Department of Internal Medicine I, Medical University of Vienna, Vienna, Austria; 4 Department of Pathology, University of Michigan, Ann Arbor, MI USA; 5 Department of Biomedical Imaging and Image-Guided Therapy, Medical University of Vienna, Vienna, Austria; 6 Center for Medical Statistics, Informatics, and Intelligent Systems, Medical University of Vienna, Vienna, Austria; 7 Department of Pathology, Medical University of Vienna, Vienna, Austria; 8 Department of Surgery, Elisabethinen Hospital Linz, Linz, Austria; 9 Department of Surgery, Landesklinikum Mistelbach, Mistelbach, Austria; 10 Department of General‑, Visceral- and Tumor Surgery, Wilhelminenspital, Vienna, Austria; 11 Department of Surgery, Kaiser Franz Josef Hospital, Vienna, Austria; 12 Department of Surgery, Landesklinikum Wr. Neustadt, Wr. Neustadt, Austria; 13 Department of General‑, Visceral- and Thoracic Surgery, Landeskrankenhaus Feldkirch, Feldkirch, Austria; 14 Department of Surgery I, Krankenanstalt Rudolfstiftung, Vienna, Austria; 15 Department of Visceral- , Transplant- and Thoracic Surgery, Medical University Innsbruck, Innsbruck, Austria; 16 Department of Surgery, Hospital Barmherzige Brüder St. Veit/Glan, St. Veit/Glan, Austria; 17 Department of Surgery and Vascular Surgery, Hanusch Hospital, Vienna, Austria; 18 Department of Hematology and Oncology, Landeskrankenhaus Leoben, Leoben, Austria

**Keywords:** Randomized biomarker trial, Response prediction, Predictive marker, Mark53

## Abstract

**Background:**

In operable esophageal cancer patients, neoadjuvant therapy benefits only those who respond to the treatment. The • Pancho trial represents the first prospective randomized trial evaluating the relevance of the mark53 status for predicting the effect of two different neoadjuvant chemotherapies.

**Method:**

Biomarker analysis was conducted using the mark53 analysis. Calculation of patient number needed was based on a 60% rate of marker positivity, deduced from the results of a phase II pilot study.

**Results:**

From 2007–2012, the • Pancho trial recruited 235 patients with operable esophageal cancer in Austria. A total of 181 patients were eligible and could be subjected to mark53 analysis and randomization. After randomizing 74 patients, the overall *TP53* mutation rate was 79%. However, due to the high prevalence of marker positivity, the number of projected patients was increased to 181 patients in order to ensure a sufficient number of marker-negative patients. After completion of the trial, the overall *TP53* mutation rate was 77.9%.

**Conclusion:**

Due to high medical need, the recruitment for the academic trial was excellent. Mark53 analysis clearly detected more mutations in the *TP53* gene as compared to the cancer-specific p53 literature. Final analysis examining the interaction between the mark53 status and the effect of chemotherapies applied in the • Pancho trial is now awaited.

## Introduction

Surgical resection is the standard therapy for operable esophageal cancer, but median overall survival is poor and only a limited number of patients in locally advanced stages are cured [1, 2]. In the recent past, the addition of neoadjuvant chemotherapy has been evaluated in clinical trials. Overall results were not consistent; however, survival benefit seemed to be restricted to those patients who responded to therapy [3–5]. The use of biomarkers promises more efficacy of treatments, identifying potential responders before chemotherapy.

Since 1989, p53 has been known as the most frequently mutated or lost gene in human cancers. In 1991, the p53 tumor suppressor gene was found to induce apoptosis. One year later, p53 was shown to maintain genomic stability and it was recognized that DNA damage is an important trigger for p53 activation [6].

Many chemotherapeutic drugs act via induction of DNA damage. This generated the hypothesis that induction of apoptosis in response to DNA damage depends on the presence of normal p53.

From 2001 to 2007, the Medical University of Vienna p53research group (http://www.p53.at) conducted a phase II pilot study to evaluate the interaction between the *TP53* genotype and the effect of standard neoadjuvant chemotherapy in esophageal cancer. Patients with operable esophageal cancer received standard regimen with 5Fluorouracil(5FU)/cisplatin as preoperative treatment. Both drugs are thought to depend on normal *TP53*. The results of this study supported the hypothesis that *TP53* could serve as potential predictive marker for response to neoadjuvant chemotherapy [7].

As a next step, the • Pancho trial was initiated in 2007. The protocol of the clinical trial was based on the “marker by treatment interaction” design and aims to evaluate the influence of the mark53 status on two different neoadjuvant treatment effects in a prospective randomized way for the first time [8].

Here we report on the completion of the • Pancho trial referring to patient recruitment, the biomarker analysis, and the standards for the central review of the primary endpoint measurements.

## Materials and methods

• Pancho was approved by the ethics committee of the Medical University of Vienna and the local ethics committees of the 13 investigational centers (EK Nr 128/2007).

The study is registered by ClinicalTrials.gov under the following identifier: NCT00525200.

### Study design

**Fig. 1:**
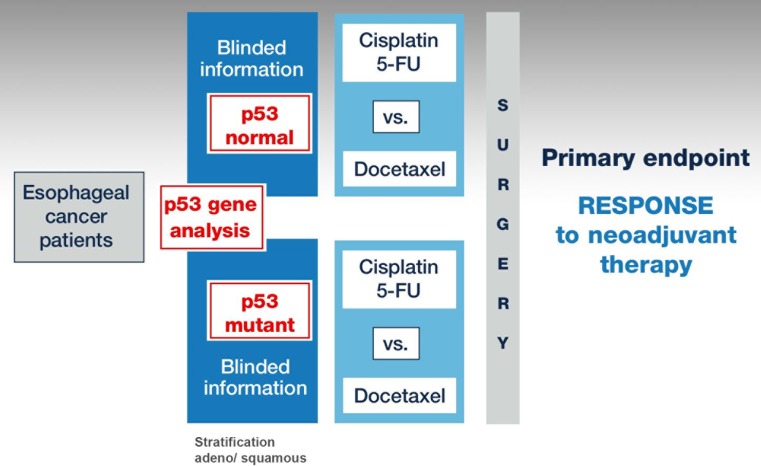
• Pancho trial design

• Pancho was designed to demonstrate in a prospective randomized clinical marker trial an interaction between the marker status and treatment effect as proposed by Sargent et al. (Fig. 1; [9]).

### Inclusion

Only patients with potentially resectable esophageal cancer, deemed fit for neoadjuvant chemotherapy and esophagectomy, were included. Early stages as well as inoperable patients were excluded. The detailed inclusion and exclusion criteria for the study have been reported elsewhere [10].

Nationwide, 13 centers participated, of which 12 centers enrolled patients.

### Randomization

The study design required knowledge of the marker status before randomization. The marker information (*TP53* status) was blinded to the investigators. Additional stratification for histological subtype (adenocarcinoma and squamous cell carcinoma) was included. Permuted block randomization was applied within mark53 status and histological subtype using the web-based Randomizer for Clinical Trials 1.8.1. at the Medical University of Vienna (https://www.meduniwien.ac.at/randomizer).

### Calculation of patient number

For designing the • Pancho trial, the prevalence of marker positivity (*TP53* mutated) was estimated to be 60%. This estimation was deduced from the results of a phase II pilot study [7]. The latter included 47 operable esophageal cancer patients who had been treated with neoadjuvant chemotherapy and whose *TP53* status was analyzed using a pilot version of the mark53 test.

A treatment difference in response rate of 60% (80% versus 20%) for both marker-positive and marker-negative subgroups was assumed from the pilot study. In order to detect this treatment difference with 82% power and a two-sided Bonferroni adjusted significance level of 0.025, at least 17 patients had to be included in each of the four arms. Based on an assumed *TP53* mutation frequency of 60%, randomization of 84 patients was originally planned.

### Marker analysis

The source material for marker analysis in the trial was DNA extracted from formalin-fixed and paraffin-embedded diagnostic tumor biopsies. The • Pancho trial serves as clinical validation for the mark53 test which provides a standardized, *TP53*-gene-specific sequencing analysis, as briefly described previously [11] (Mark53 Ltd. Vienna, Austria; https://www.mark53.com).

The marker analysis was performed centrally in the certified laboratory of the Medical University of Vienna p53research group located at the Department of Surgical Research, Medical University of Vienna.

## Results

### Recruitment

**Fig. 2:**
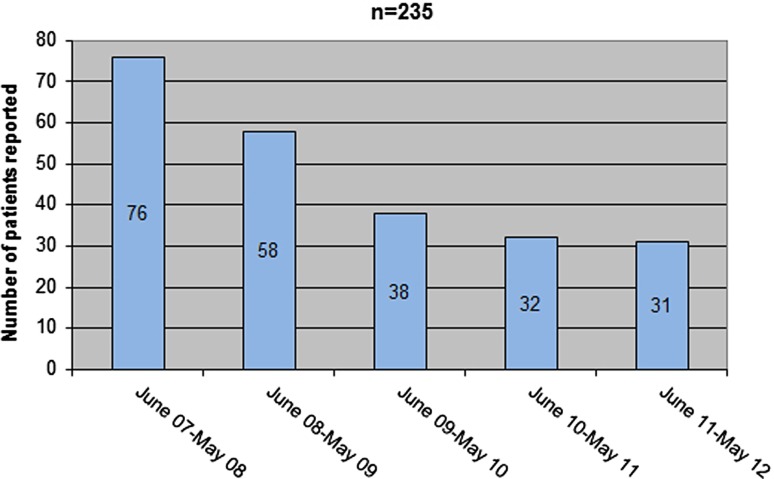
• Pancho patients reported per year

Patient recruitment started in June 2007 and was successfully completed after 5 years in May 2012 (Fig. 2).

In total, 235 patients from 12 Austrian centers were registered to the trial (Table 1).

**Table 1:** • Pancho patients reported and randomized per center

Pancho centers	Principal investigators^a^	Patients reported	Patients randomized
Medizinische Universität Wien	Univ. Prof. Dr. Johannes Zacherl	123	82
Wilhelminenspital, Wien	Prim. Univ. Prof. Dr. Karl Glaser	17	13
Kaiser Franz Josef Spital, Wien	Prim. Univ. Prof. Dr. Josef Karner	13	12
Krankenanstalt Rudolfstiftung, Wien	Prim. Univ. Prof. Dr. Rudolf Roka	6	6
Hanusch Krankenhaus, Wien	Prim. Dr. Michael Hold	3	3
Landesklinikum St. Pölten, NÖ	OA Dr. Ronald Zwrtek	20	16
Landesklinikum Wiener Neustadt, NÖ	Prim. Univ. Prof. Dr. Friedrich Längle	10	10
Krankenhaus der Elisabethinen Linz, OÖ	Prim. Univ. Prof. Dr. Reinhold Függer	20	20
Landeskrankenhaus Leoben, Steiermark	Univ. Prof. Dr. Felix Keil	2	2
Krankenhaus der Barmherzigen Brüder St. Veit, Kärnten	Prim. Univ. Prof. Dr. Jörg Tschmelitsch	5	3
Medizinische Universität Innsbruck, Tirol	a.o. Univ. Prof. Dr. Dietmar Öfner	6	4
Landeskrankenhaus Feldkirch, Vorarlberg	Prim. Univ. Prof. Dr. Etienne Wenzl	10	10
*Sozialmedizinisches Zentrum Ost, Wien*	*OA Dr. Nikolaus Hölbling*	*0*	*0*
**Total**	–	**235**	**181**

^a^Principal investigators and their positions at the time of initiation of the trial

Of these, 54 had to be excluded:

- Early stage cancer (cT1; *n* = 5)
- Metastases or second primary cancer (*n* = 20)
- Other tumor therapy (*n* = 12)
- No consent (*n* = 4)
- Withdrawal of agreement (*n* = 6)
- Medical condition (*n* = 3)
- Other reasons (*n* = 4)

Mark53 analysis and randomization was performed in 181 eligible patients. Histological subtype was used for stratification. Adenocarcinoma was found in 57% (103/181), and squamous cell carcinoma in 43% (78/181).

### Results from biomarker analysis

After having randomized 74 patients, a mutated mark53 status was revealed to be present in 79% of the patients. This was clearly above the estimated prevalence of 60%, which served as basis for the calculation of the patient number needed in the • Pancho trial.

Thus, in December 2008, it was decided to increase the overall sample size. This was done in an amendment to the study protocol, approved by the ethics committee. Based on the high prevalence of marker positivity, instead of the projected 84 patients, 181 patients had to be randomized to the • Pancho trial in order to recruit a sufficient number of marker-negative patients.

In 2012, the • Pancho trial was completed with the inclusion of 141 marker-positive and 40 marker-negative patients. This corresponds to a final TP53 mutation rate of 77.9%, with a 95% confidence interval of 71.3 to 83.3%.

### Validation of endpoint measurements

Primary endpoint of the • Pancho trial is response to treatment as measured by radiological and pathological response.

Disease-free survival (DSF) and overall survival (OS) serve as secondary endpoints.

Central independent reviews were requested to ensure standardized measurements of the primary endpoints (radiological and pathological response).

#### The radiological central review

CT scans of the neck, chest, and abdomen were performed at the time of diagnosis and after neoadjuvant chemotherapy (=preoperatively). Scans were obtained after injection of i.v. contrast in the arterial and portal venous phase. Since the CT scans were performed by the study centers, the protocols varied to some degree according to local circumstances. Scans were centrally reevaluated in terms of tumor size and clinical tumor stage. Marker status and clinical data as well as the original CT findings of the study centers were blinded to the reviewing radiologists. All scans were read by two radiologists in consensus (6 and 22 years of experience).

Tumor size was assessed by the maximal longitudinal extension and the maximal wall thickness. Variations between pre- and post-chemotherapy measurements were described as increased, decreased, or stable tumor length.

T and N stage were analyzed as described previously [12]. Clinical tumor stage was assessed according to the TNM classification of the Union for International Cancer Control (UICC), 6^th^ edition [13]. Variations between pre- and post-chemotherapy in T and N category were described as increase, decrease, or stable tumor stage.

#### The pathological central review

Hematoxylin–eosin-stained slides of the surgical specimens were provided by pathological institutes of the study centers. Slides were centrally reevaluated regarding the pathological staging and tumor regression. Marker status and clinical data as well as the original pathological findings of the study centers were blinded to the reviewing pathologist.

Pathological staging was based on the American Joint Committee on Cancer criteria (AJCC), 7^th^ edition [14].

For assessment of tumor regression, two systems based on the percentage of viable tumor cells in relation to the macroscopically identifiable tumor bed in the previous site of the tumor were used. In the four-tier Chirieac system, the following categories are used: “1” represents complete regression, “2” = 1 to 10% viable tumor cells, “3” = 11 to 50%, and “4” ≥ 50% or without signs of treatment effect [15]. In the modified three-tier Chirieac system, “0” represents 0% of viable tumor cells, “1” ≤ 50%, and “2” > 50% [16].

#### Overall survival and disease-free survival

The last patient was randomized in May 2012. In November 2017, all patients reached the 5‑year follow-up.

## Discussion

The population of Austria consists of 8.7 million people. Per year, 160–180 patients suffering from esophageal cancer in an operable stage of disease are diagnosed in this country. Roughly 100 of them present with a locally advanced stage and therefore qualify for neoadjuvant therapy (http://www.statistik-austria.at/web_de/statistiken).

The • Pancho trial enrolled 235 operable esophageal cancer patients within 5 years (2007–2012) in Austria. Thus, almost 50% of all potentially suitable patients within Austria were registered to the clinical trial, ranging from 76% in the first year to 31% in the fifth year (Fig. 2). Given that it was an academic trial, the study did not budget for patient fees or case compensation. Nevertheless, the recruitment was very successful, which we attribute to the high medical need and the marked potential improvement for treatment outcome promised by the use of the biomarker.

By randomization of 181 operable esophageal cancer patients to neoadjuvant chemotherapy, the • Pancho cohort represents one of the largest prospectively randomized collections of preoperatively treated esophageal cancer patients thus far [17]. Furthermore, this trial represents the first prospective randomized validation of a biomarker potentially predicting the effect of neoadjuvant chemotherapy.

The prevalence of marker positivity is mandatory information for designing a biomarker trial as it is directly related to the number of patients needed to detect the difference sought. At the time of initiation of the • Pancho trial in 2007, the most important p53 databases reported a 40% prevalence for* TP53* mutations in esophageal cancer (IARC TP53 Mutation Database, R12 release, Nov 07, http://www-p53.iarc.fr; UMD_TP53 Mutation Database, 2006, http://p53.free.fr) [18].

In 2007, we reported a 66% *TP53* mutation rate in a phase II pilot study including 47 operable esophageal cancer patients analyzed with an early version of the mark53 test [7].

Following the analysis of 181 operable esophageal cancers with the standardized mark53 test, the completed • Pancho trial ultimately reports a mutation prevalence of 77.9%.

The mark53 test was developed by the Medical University of Vienna p53research group to allow a virtually complete detection of genetic deviations of the *TP53* gene. The method of TP53-gene-specific sequencing has since been patented and is registered under the name mark53® test. In recent years, the p53research group has validated the mark53 test in a number of clinical trials [7, 11, 19, 20]. It was shown that mark53 analysis provided a higher cancer-specific mutation rate when compared to the p53 literature, and most importantly, a significant interaction between the mark53 status and effect of standard treatments in different cancers was consistently demonstrated. These findings suggest that *TP53* mutations might be underreported in the literature. If *TP53* mutations are in fact underreported, this may obstruct future evaluations analyzing whether the marker is of prognostic or rather predictive nature.

The next step of the • Pancho trial will be to clinically validate the mark53 test in this prospective randomized study looking for the interaction of the mark53 status with the effect of two different treatments.

## Appendix

### List of investigators

The following study team members (Principal Investigators, Sub-Investigators, Study Coordinators, Study Nurses, Pathologists, Radiologists, and others) were involved in the • Pancho study at sites:

- *Medical University Vienna*: D. Kandioler, J. Zacherl, M. Hejna, S. Schoppmann, M. Gnant, U. Pluschnig, I. Kührer, W. Klepetko, G. Prager, M. Riegler, C. Aigner, B. Teleky, S. Kappel-Latif, C. Bichler, B. Wolf, G. Werba, C. Sandurkov, D. Tamandl, A. Ba-Ssalamah, M. Uffmann, F. Wrba, M. Mittlböck, B. Niederle, R. Jakesz, F. Mühlbacher, J. Friedl, R. Kain, L. Brammen
- *Wilhelminenspital, Vienna*: K. Glaser, F. Berger, S. Brugger, S. Sporn, K. Strasser-Weippl, R. Fortelny, M. Essenther, A. Chott
- *Kaiser Franz Josef Hospital, Vienna*: J. Karner, S. Thalhammer, M. Klimpfinger
- *Krankenanstalt Rudolfstiftung, Vienna*: R. Roka, M. Schermann, M. Kees-Belyus, V. Sagaster, T. Grünberger, E. Bonner
- *Hanusch Hospital, Vienna*: M. Hold, M. Bernhart, S. Roka, C. Österreicher, V. Riegler, A. Nader, J. Haller
- *Landesklinikum St. Pölten*: R. Zwrtek, P. Götzinger, M. Pober, T. Schenk, W. Guggenberger, R. Sedivy, M. Kitzwögerer, G. Heinz, M. Thür
- *Landesklinikum Wr. Neustadt*: F. Längle, I. Viragos-Toth, E. Frcena, H. Pourebrahim, E. Kristandl, W. Stiglbauer
- *Elisabethinen Hospital, Linz*: R. Függer, F. Tomaselli, S. Metz, F. Moinfar
- *Landeskrankenhaus Leoben*: F. Keil, U. Kastner, H. Rabl, C. Tinchon, V. Odelga, N. Rapp, N. Eberhard, H. Kainz, M. Maderdonner, G. Leitner
- *Hospital Barmherzige Brüder St. Veit*: J. Tschmelitsch, T. Eberl, H. J. Neumann, H. Weiß, J. Mühlmann, K. Weeber, G. Danko
- *Medical University Innsbruck*: D. Öfner, G. Mühlmann, M. Zitt, H. Maier, B. Heinke, W. Eisterer, N. Bergmann, E. Dablander, G. Mikuz, A. Brunner
- *Landeskrankenhaus Feldkirch*: E. Wenzl, A. Haid, K. Ammann, M. Knauer, A. Lang, B. Hartmann, M. Lercher-Lueger, F. Offner, B. Aberer
- *Datatechnology, Vienna*: M. Hudec
- *CTM Clinical Trials Management, Vienna*: M. Hohlagschwandtner, W. Trabe, P. Merz, V. Kadlecek, U. Smetana, D. Veit, T. Veit
- *Pathology institute, Vienna*: D. Kerjaschki, O. Braun, R. Kuzmits, D. Kosak, W. Adolf, T. Kessler, C. Wüstinger, N. Neuhold
- *Pathology institute, NÖ*: F. Beer, C. Freibauer, S. Naude, O.M. Braun, M. Mostegel, F. Pantucek
- *Pathology institute, OÖ*: J. Feichtinger, W. Sega, H. Gogl, W. Höbling, R. Silye, E. Beck
- *Pathology institute, Graz*: H. Denk, G. Höfler, K. Lichtenegger
- *Pathology institute, Klagenfurt*: H. Rogatsch, S. Gallowitsch
- *Pathology institute, Salzburg*: O. Dietze
